# Navigating postgraduate research during the COVID-19 pandemic: A scoping review

**DOI:** 10.4102/curationis.v45i1.2373

**Published:** 2022-09-21

**Authors:** Patricia Y. Mudzi, Witness Mudzi

**Affiliations:** 1 Department of Nursing, Faculty of Health Sciences, University of Pretoria, Pretoria, South Africa; 2 Center for Graduate Support, University of the Free State, Bloemfontein, South Africa

**Keywords:** postgraduate, research, students, challenges, opportunities, COVID-19

## Abstract

**Background:**

The worldwide coronavirus disease 2019 (COVID-19) pandemic compelled higher education institutions and postgraduate students (master’s and PhD) to rethink their research designs, as alert level restrictions affected data collection.

**Objectives:**

To identify and map out the breadth of evidence available on the challenges and opportunities for conducting postgraduate research during the COVID-19 pandemic.

**Method:**

A scoping review was conducted in CINAHL, MEDLINE (EBSCO), SCOPUS and PubMed databases. The authors independently began by screening the titles, the abstracts and full texts. Duplications were removed during the title and abstract screening by exporting them into EndNote.

**Results:**

The search identified 463 documents, and 12 were included in the final review. The documents were studies commentaries, letters (*n* = 1) from India and guidelines from countries across the globe. The common themes that were reported on the challenges of conducting postgraduate research were the inability to collect data, the digital divide and changes in study designs.

**Conclusion:**

The review reports on the opportunities and challenges in conducting postgraduate research during the COVID-19 pandemic. Despite the limited evidence on the opportunities of conducting postgraduate research, most postgraduate research stalled because of COVID-19 restrictions. There is a need for more literature to explore further postgraduate research opportunities during COVID-19 and beyond.

**Contribution:**

The focus on the challenges and opportunities in conducting postgraduate research during the COVID-19 pandemic may assist in the development of strategies needed to mitigate the effects of this and future pandemics.

## Introduction

The broader impact of the coronavirus disease 2019 (COVID-19) pandemic on postgraduate research in South Africa is concerning. This impact has been felt in higher education institutions and includes supervising postgraduate students’ research, meeting research output targets, submitting new grants to secure the next cycle of research and meeting research funding and project deliverables (Hedding et al. 2020:7). The pandemic has affected ongoing clinical trials that are evaluating interventions aimed at preventing or treating diseases other than COVID-19 (Fleming, Labriola & Wittes 2020:33). In addition, conducting postgraduate research during the pandemic has given rise to practical and ethical challenges as it became apparent that there was a need to strike a balance between generating new knowledge and the realities of the pandemic.

The pandemic forced postgraduate students to change their research methodologies, plans and scope (Motala & Menon 2020:80). Other postgraduate students had to request an extension of their research projects because of difficulties collecting participant data courtesy of lockdown restrictions (Motala & Menon 2020:80). Funded students faced a risk of having to fund their studies as they passed the deadlines because of the inability to collect data under lockdown regulations (Van Tienoven et al. 2022:1).

Postgraduate students transitioned from research that involved face-to-face interaction to conducting alternative research activities such as writing proposals, grants and protocols; simulation and modelling; and statistical analysis (Persky et al. 2020:1). Postgraduate research focusing on the elderly population had to be halted because the elderly were among the most vulnerable to COVID-19 (Oviedo et al. 2021:1). In some cases, postgraduate students had to completely change their research methodologies (Van Tienoven et al. 2022:1) from the ones for which they had received ethical clearance. This led to increased time to completion of their research projects. Nonetheless, the change in research design also presented an opportunity for challenging main research methods typically used in a discipline, encouraging both the postgraduate student and their supervisors to learn new methodologies (Motala & Menon 2020). In the same vein, postgraduate students viewed the adoption of online interviewing instead of face-to-face as facilitating increased participant recruitment while cutting costs (King et al. 2022:671). The skills acquired by the postgraduate students as they adapted their research methods positively encouraged awareness of their potential in adapting to new research methods (King et al. 2022:671).

The pandemic offered postgraduate students opportunities to develop in the use of technology. However, it can be argued that by design, technology excludes the disadvantaged, resulting in inequalities and digital exclusion that may impact research participants, students and their supervisors (Dawood & Van Wyk 2021:1). Postgraduate students reported that since the lockdown, they had more time to write manuscripts. At the same time, some indicated that the lockdown heightened their anxiety and limited their research productivity. Such complexities show that we live in a complex world with a plethora of real-world problems. Hence, postgraduate students’ experiences when conducting research cannot be generalised (Hedding et al. 2020:7).

A commentary by Aydemir and Ulusu (2020:428) stated that postgraduate students could manage their time effectively by widening their knowledge base through reading literature, consulting supervisors for thesis or dissertation support and writing papers, thus turning the COVID-19 crisis into an opportunity for academic growth. This scoping review aimed to identify and map out the breadth of evidence available on the challenges and opportunities of conducting postgraduate research during the COVID-19 pandemic.

## Methodology

The review was guided by the Arksey and O’Malley (2005:1) scoping review framework, which has the following stages: (1) identifying the research question; (2) identifying relevant studies; (3) study selection; (4) charting the data; and (5) collating, summarising and reporting the results. The Preferred Reporting Items for Systematic Review and Meta-analysis Protocols (PRISMA-P) 2020 (Page et al. 2021:1) flow chart was utilised to report how the articles found from the search were sorted.

### Stage 1: Identifying the research question

The main objective of the review was to identify and map out the breadth of evidence available on the challenges and opportunities of conducting postgraduate research during the COVID-19 pandemic. Therefore, the broad research question for the review was, ‘What are the challenges and opportunities in conducting postgraduate research during the COVID-19 pandemic?’

#### Definition of key concepts

*Challenges* are new or difficult tasks that test someone’s ability and skill (Cambridge University Press 2022a). In this review, challenges refer to the difficulties or struggles encountered in conducting postgraduate research.

*Opportunities* are times when a particular situation makes it possible to do or achieve something (Cambridge University Press 2022b). In relation to the review, opportunities are situations that made conducting postgraduate research possible.

*Postgraduate research* includes research connected with further studies undertaken at a university after students receive their first degree (HarperCollins 2022). In relation to this study, it refers to research that postgraduate students undertake.

A *pandemic* is a disease that spreads and affects a large number of people (Cambridge University Press 2022c). Concerning the review, it refers to the severe acute respiratory syndrome coronavirus 2 (SARS-CoV-2) virus.

*Coronavirus disease 2019* (COVID-19) is an infectious disease caused by the SARS-CoV-2 virus (WHO 2020). In relation to this study, it is the virus that has spread worldwide and influenced postgraduate research.

#### Justification of the need for the review

The review may assist in highlighting challenges and opportunities in conducting postgraduate research during the COVID-19 pandemic. Knowledge and findings of these factors may be used to assist in the development of innovative ways of conducting research during the COVID-19 pandemic and beyond.

### Stage 2: Identifying relevant studies

A literature search was conducted in multiple electronic databases: CINAHL, MEDLINE (EBSCO), SCOPUS and PubMed from 2019 to 2022. Grey literature from OpenGrey.eu and Greylit.org websites was sought. The following search terms were used in various combinations, based on Boolean phrases, to search for published articles that met the inclusion criteria for the review: ‘Opportunities or Challenges’ and ‘COVID-19 and Postgraduate and Research’, ‘Postgraduate research and COVID-19’, ‘Postgraduate students or Master’s or PhD or Doctoral and Research and COVID-19’.

The study used the population, concept and context (PCC) framework to identify the main concept in the review question and inform the search strategy. Table 1 illustrates the PCC framework (Peters et al. 2020:2119).

**TABLE 1:** Population, concept and context framework.

PCC element	Description
Population	Postgraduate students (Master’s and PhD)
Concept	Challenges, opportunities, research, COVID-19, pandemic
Context	Higher education, hospitals, communities, colleges of higher education

*Source:* Adapted from Peters, M.D.J., Godfrey, C., McInerney, P., Munn, Z., Tricco, A.C. & Khalil, H., 2020, ’Chapter 11: Scoping Reviews (2020 version)’, in E. Aromataris & Z. Munn (eds.), *JBI Manual for Evidence Synthesis*. https://doi.org/10.46658/JBIMES-20-12

PCC, population, concept and context.

### Stage 3: Study selection

#### Inclusion criteria

Studies were included in the review if they were published between 2019 and 2022. This publication range was used because the COVID-19 outbreak was first reported in 2019, and it is still ongoing, having been declared a pandemic. Scholarly, peer-reviewed, full-text evidence-based studies and grey and empirical literature were included. Literature published in English and conducted in higher education, hospitals, community and higher education colleges were also included. Qualitative, quantitative and mixed methods studies were included in the review.

#### Exclusion criteria

Studies were excluded if they were non-English and published before 2019. Evidence not related to challenges and opportunities of postgraduate research was excluded.

**FIGURE 1:**
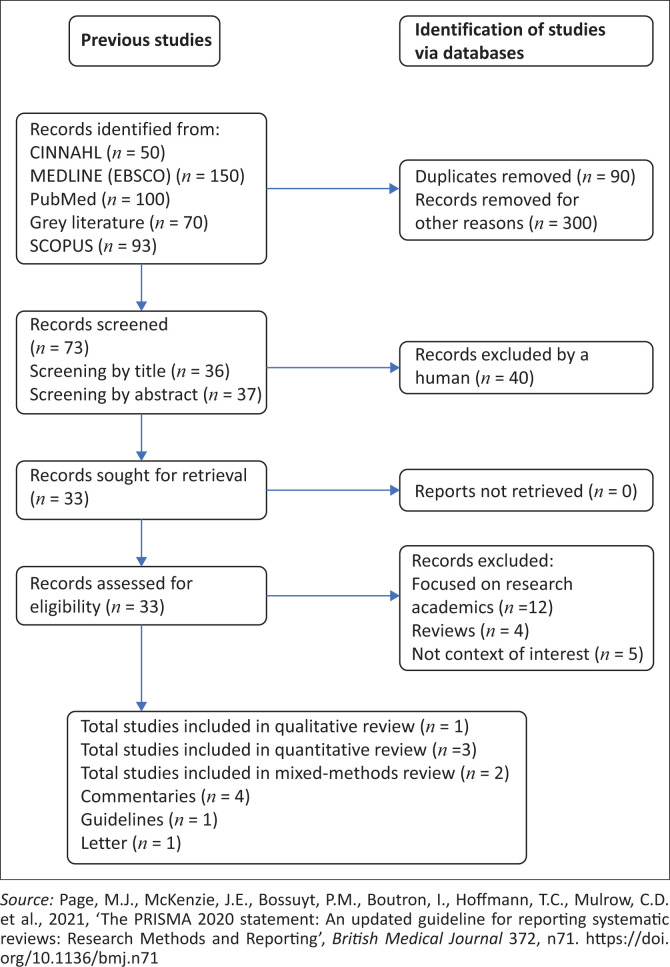
PRISMA 2020 flow diagram of studies included in the review.

The literature search and review results are reported in the PRISMA flow diagram (Figure 1). The literature on the various online platforms identified 463 full-text documents, including grey literature; 90 duplicates were removed; another 300 documents were removed for other reasons such as ineligible study design and ineligible population; 73 documents remained. Initial screening of the titles resulted in retaining 36 documents, and screening by abstract retained 37 documents; 40 were excluded by human factor because their titles were not relevant to the review. There were 33 documents retrieved and assessed, of which a further 21 documents were removed for the following reasons: focused on research academics (*n* = 12), reviews (*n* = 4) and not the context of interest (*n* = 5), as shown in Figure 1. Therefore, to answer the research question, 12 documents that met the inclusion criteria were included in the review (Table 2 and Table 3).

**TABLE 2:** Summary of studies included in the scoping review following full-text review.

Author(s), year, Country	Study aim or purpose	Study design and sampling technique	Population and sample features	Method of data collection	Key findings related to challenges	Key findings related to opportunities
Rafat and Khan (2021) India	To assess challenges faced by postgraduate obstetrics and gynecology residents in doing research during the COVID-19 pandemic	Quantitative cross-sectional study Sampling – purposive	21 female postgraduate students	Questionnaires	Inability to collect data 52.4% stated that they had inadequate time for research Limited time to interact with thesis supervisors Cancellation of face-to-face seminars and conferences Insufficient thesis mentoring workshops Limited access to the Internet for research purposes	Postgraduate students utilised the pandemic time for scholarly purposes and others (88%) reviewing the literature
Pyhältö et al. (2022) Finland	To explore the influences of the pandemic on PhD candidates’ progress	Mixed methodsdesign Sampling – not indicated	768 PhD candidates	Questionnaires Interviews	Interrupted recruitment of study participants Limted postgraduate supervision Cancelled conferences and research visits Exclusion from the research community and Reduced research engagement	None reported
Dong et al. (2022) China	To investigate the impact of COVID-19 social distancing on medical research from the perspective of postgraduate students	Quantitative cross-sectional Sampling – not indicated	468 postgraduate medical students	Questionnaires	Social distancing halted or hindered data collection and laboratory work for research purposes	Staying at home during the pandemic measures enabled postgraduate students to spend more time on article writing
Van Tienoven et al. (2022) Begium	To investigate the extent of PhD students’ satisfaction with supervisor and research support	Longitudinal cohort Quantitative Sample – not indicated	694 PhD students	Questionnaires	PhD students altered their research designs Research projects were extended Interrupted data collection Postponed research Redesigning research Laboratory research was scaled down to take into account social distancing regulations	Postgraduate students who had finished collecting data were less affected than those still collecting data Opportunity to familiarise oneself with online technology More time for article writing
King et al. (2022) United Kingdom	To explore the views of psycho-oncology postgraduate research students on the impact of the COVID-19 pandemic on learning and research	A cross-sectional qualitative survey	19 postgraduate psycho-oncology researchers	Online survey with open-ended questions	Research projects’ timelines increased. Slowed research progress because of the inability to collect data for research	Pandemic offered an opportunity for adapting research methods Online data collection increased recruitment and limited costs
Hofmeyr, Price and Myres (2021) South Africa	To understand how COVID-19 has affected postgraduate business school students	Mixed methods	853 postgraduate business school students	Questionnaire Open-ended questions	Students had to change the research design	71.7% of postgraduate students perceived themselves as resilient because of the pandemic

*Source*: Roman, N.V. & Frantz, J.M., 2013, ‘The prevalence of intimate partner violence in the family: A systematic review of the implications for adolescents in Africa’, *Family Practice* 30(3), 256–265. https://doi.org/10.1093/fampra/cms084

**TABLE 3:** Summary of guidelines, commentaries and letters included in the scoping review.

Authors, year, country	Type of document	Aims, objectives, purpose theme	Target population	Challenges	Opportunities
Greeff (2020) South Africa	Guideline	To guide postgraduate students and academic researchers on conducting qualitative research during the lockdown and physical distancing	Postgraduate and academics	Uncertainty of the security of online platforms during data gathering Difficulty in engaging with the community and entry into the community The richness of observation while interviewing is lost when collecting data online Digital divide, high cost of airtime and connectivity	Community engagement and community entry could be done through online platforms (Skype, Zoom)
Hedding et al. (2020) South Africa	Commentary	Impact of COVID-19 on research and suggestions on mitigation	Postgraduate students, researchers, undergraduate students	Funded students had difficulty completing their research projects in time, resulting in the withdrawal of funds Difficulty in meeting the research output targets	Postgraduate students had adequate time during the pandemic to mine older data sets and extract information from large online data sets Postgraduate students could conduct research entirely online or undertake research that is conceptual in nature
Pepper and Burton (2020) South Africa	Commentary	The importance of research and strategies that will need to be implemented to ensure that research activities continue	Postgraduate students, researchers and scientists	Restarting some experiments as biological specimens may no longer be viable because of the lockdown restriction Instruments may malfunction Supplies for research work are likely to be delayed because of slowed deliveries as suppliers respond to a wave of orders	None reported
Persky et al. (2020) United States	Commentary	To describe how pharmaceutical sciences respond to the current COVID-19 pandemic and anticipate impact moving forward by envisioning future best practices for postgraduate students	Postgraduate pharmacy students	Digital divide Active recruitment of patients into clinical trials was also placed on hold	Research activities shifted to writing protocols and manuscripts and analysis of data Some postgraduate students were offered the opportunity to be redeployed to research efforts focusing on COVID-19
Aydemir and Ulusu (2020) Turkey	Commentary	To turn COVID-19 crisis into an opportunity	PhD students	Keeping a balance between laboratory research and reading literature to gain knowledge about scientific developments was a challenge	Opportunity to widen knowledge in and read more articles related to their area of research Redesigning new experiments, writing and analysing data of previous experiments
Wadgave (2021) India	Letter	Impact of COVID-19 on academic research in India	Postgraduate students	Lockdown made it difficult for postgraduate students to complete their research within the stipulated time frame Postgraduate students may resort to unethical or fraudulent research activities to complete their research, compromising the scientific evidence’s credibility and validity	Not reported

### Stage 4: Charting of data

The characteristics of the studies extracted consisted of key information such as author(s), year, country, study aim or purpose, study design and sampling technique, population and sample features, method of data collection and key findings related to the scoping review question. Both authors extracted the studies. The authors assessed whether the title and abstracts met the PCC search strategy. This was done by firstly screening the titles and exporting them into EndNote (Clarivate Plc, London, United Kingdom) with assistance from the librarian. This process eliminated the duplicates. Thereafter, the abstracts of the included titles were obtained. The authors independently screened the abstracts and the full-text articles and obtained full texts of the articles that met the inclusion criteria. Disagreements were collaboratively resolved through peer discussion before including the articles for further consideration (see Table 2).

The review identified studies that reported on the challenges and opportunities of postgraduate research from India, Finland, Belgium, China, the United Kingdom and South Africa.

The summary (Table 3) of guidelines (*n* = 1), commentaries (*n* = 4) and letters (*n* = 1) were included in the scoping review.

### Stage 5: Collating, summarising and reporting results

This scoping review aimed to identify and map out the breadth of evidence available on the challenges and opportunities of conducting postgraduate research during the COVID-19 pandemic. The data will be presented descriptively.

## Ethical considerations

The study adhered to ethical standards without direct contact with human or animal subjects.

## Results

The studies identified were from: India (*n* = 1), Finland (*n* = 1), Belgium (*n* = 1), China (*n* = 1), the United Kingdom (UK) (*n* = 1) and South Africa (*n* = 1). Commentaries from South Africa (*n* = 2), the United States (US) (*n* = 1) and Turkey (*n* = 1) and a letter (*n* = 1) from India were identified and included in the review. The common themes identified from the review were the collection of data, change in study designs and the digital divide. Other challenges that were identified were limited face-to-face seminars and conferences for postgraduates to exchange ideas on research (Rafat & Khan 2021:S77), supervising postgraduate students, meeting research output targets (Hedding et al. 2020:7) and increased timelines for research projects, resulting in termination of funding (Van Tienoven et al. 2022:1). The pandemic also presented opportunities for postgraduate students to learn new research methodologies, as they had to adapt their research designs to be able to collect data from participants.

## Challenges and opportunities in conducting postgraduate research during the COVID-19 pandemic

### Collection of data

According to studies conducted in India (Rafat & Khan 2021:S77), Finland (Pyhältö et al. 2022), the US (Persky et al. 2020:1), Belgium (Van Tienoven et al. 2022:1), South Africa (Hofmeyr et al. 2021:1) and the UK (King et al. 2022:671), postgraduate research was hampered by the inability to collect data from participants because of COVID-19 regulations of physical distancing. Postgraduate students had to halt their studies, as their study participants were from vulnerable populations (e.g. elderly) (Oviedo et al. 2021:1). Contrary to the findings by Oviedo et al. (2021:1), Aydemir and Ulusu (2020:428) reported that some postgraduate students had the opportunity to write articles, analyse data from previous experiments and widen their scientific horizons in their fields of research. About 88% of the postgraduate students felt that the pandemic provided them with adequate time for a literature review of their research projects (Aydemir & Ulusu 2020:428).

The security of online platforms during data gathering was reported as a challenge in conducting research during the pandemic (Greef 2020:1), given the online systems’ vulnerability to hacking. Additionally, the richness of participant observation while conducting face-to-face interviews was lost when using online platforms for data collection methods (Greef 2020:1). In a commentary by Pepper and Burton (2020:1), research involving the use of biological materials had to be put on hold because of the alert level restrictions rendering them unviable and costly to replace; postgraduate students whose research involved community engagement and making use of community entry gatekeepers struggled to gain entry into communities because of the alert level restrictions (Greef 2020:1).

### Change in study designs

Some postgraduate students had to change designs to be able to collect data from participants (Hofmeyr et al. 2021:1; King et al. 2022:671; Van Tienoven et al. 2022:1). Nonetheless, some postgraduate students adapted their research to be conceptual in nature, while others changed their research designs to include online data collection (King et al. 2022:671; Van Tienoven et al. 2022:1).

### Digital divide

Despite some postgraduate students having technology that enabled them to conduct research during the pandemic, disadvantaged communities were excluded (Greeff 2020:1; Persky et al. 2020:1). The digital exclusion may have impacted research participants as well during data collection.

## Discussion

The pandemic had an impact on conducting postgraduate research; hence, there is a need to understand the challenges and opportunities that arose during the pandemic. This scoping review reports on the current literature on challenges and opportunities for conducting postgraduate research in the COVID-19 era.

The findings of this review revealed common themes regarding the opportunities and challenges of postgraduate research. The themes were the collection of data, the digital divide and changes in study designs. The inability to recruit participants and/or follow them up was reported by various authors (Rafat & Khan 2021: Van Tienoven et al. 2022:1). This was attributed to the COVID-19 pandemic restrictions which prohibited face-to-face interactions with participants. Consequently, most postgraduate research stalled. However, in a study conducted in China, postgraduate students benefited from stay-at-home measures, as they spent most of their time writing manuscripts (Dong et al. 2022:1), mining older data sets and extracting information from large online data sets (Hedding et al. 2020:7). Limited face-to-face seminars and conferences where postgraduates exchange ideas on research were reported as one of the challenges that postgraduates students faced during the pandemic (Rafat & Khan (2021:S77). The limited interaction left some postgraduates students demotivated (Pyhältö et al. 2022). In a study conducted in India, postgraduate students had difficulty maintaining communication with their thesis supervisors who were meant to support and guide them through their research projects (Rafat & Khan 2021:S77).

Postgraduate students who had planned electronic data collection or had already collected data were minimally affected by the pandemic. The mandatory stay-at-home measures enabled them to have more time to write up their research projects (Van Tienoven et al. 2022:1). However, postgraduate students who had to change their research designs to include online data collection had to take into account the availability of the Internet, and appropriate hardware and software for data collection may be in short supply in resource- constrained communities (Greef 2020:1; Hofmeyr et al. 2021). The pandemic also allowed postgraduate students to familiarise themselves with online data collection and adapt their research methods (King et al. 2022:671).

Postgraduate students undertaking research involving biological materials were affected the most by the lockdown restrictions, which emphasised mandatory staying at home. Their research ran a risk of being restarted, as biological material may become nonviable. Expensive supplies were likely to be slowly delivered as suppliers responded to a wave of orders (Pepper & Burton 2020:1). The inability of postgraduate students to collect data because of the COVID-19 restrictions may have tempted some of them to resort to unethical or fraudulent research activities such as data manipulation or fabrication to complete their research, which compromises the scientific integrity of research (Wadgave 2021:1).

In South Africa, the pandemic impacted the supervision of postgraduate students and the meeting of research output targets (Hedding et al. 2020:7), as most of them could not meet their project deliverables because of alert level restrictions. Additionally, the strict time frames for research completion that some funding agencies imposed made it difficult for postgraduate students to continue with their research because of the limited number of years of student financial support (Hedding et al. 2020:7). This impeded the postgraduate students’ research progress.

## Strengths and limitations

The review was limited to postgraduate research and did not consider the challenges and opportunities of COVID-19 for renowned researchers. The review also considered studies written in English, and there may be similar studies written in other languages.

## Conclusion and recommendations

The findings from the review reveal that the themes identified in the challenges of postgraduate research were the collection of data, the digital divide and changes in study designs. However, there is limited literature on the opportunities to conduct postgraduate research; hence, more studies must be undertaken focusing on enhanced research methods during pandemics and beyond.
